# The influence of sports technology device usage on middle-aged health anxiety: chain mediation effects of health control perception and self-efficacy reconstruction

**DOI:** 10.3389/fpubh.2025.1687208

**Published:** 2026-01-12

**Authors:** Feixuan Li, Jiahui Peng, Li Cao, Deqiao Zhou

**Affiliations:** 1 School of Physical Education and Sport Science, Qufu Normal University, Jining, China; 2 School of Physical Education, Shandong Normal University, Jinan, China; 3 School of Physical Education, Shandong University, Jinan, China

**Keywords:** sports technology devices, middle-aged cohort, health anxiety, buffering mechanism, health control perception, self-efficacy reconstruction, chain mediation

## Abstract

**Background:**

The accelerating aging of the population and the digital transformation of health management have created new challenges for middle-aged cohorts (MACs). These individuals face escalating health anxiety (HA) stemming from declining physiological functions and compounded social role pressures. Sports technology devices (STDs) offer novel behavioral intervention pathways through real-time monitoring and data feedback. However, their buffering mechanisms (BMs) against HA and the associated psychological pathways remain insufficiently explored. This study examines the chain mediation relationship involving health control perception (HCP) and self-efficacy reconstruction (SER). Our primary objective is to delineate the psychological pathways linking STD usage to HA among middle-aged individuals. This investigation provides a novel theoretical contribution to the development of targeted digital health interventions.

**Methods:**

We conducted a cross-sectional survey with 930 middle-aged participants. The study employed validated scales to measure sports technology usage, health anxiety, health locus of control, and self-efficacy. To examine the psychological mechanisms, we performed a chain mediation analysis using bootstrap regression with 5,000 iterations while controlling for key demographic factors, including age, sex, and income.

**Results:**

STD usage intensity showed a significant negative correlation with HA (*r* = −0.817, *p* < 0.01), demonstrating a total effect size of −0.7646 (95% CI [−0.7995, −0.7297]). This accounted for 66.7% of the observed anxiety variance. Both HCP (*β* = −0.715) and SER (*β* = −0.624) exhibited independent mediation effects. Notably, the chain mediation pathway explained 31.12% of the total association. The largest contributing pathway was “STD → HCP → SER → HA” (effect = −0.1438, proportion = 18.81%).

**Conclusion:**

STD usage alleviates HA among middle-aged individuals through a sequential “data-driven control enhancement → efficacy accumulation → anxiety mitigation” relationship. The core process involves reducing health uncertainty via real-time monitoring while rebuilding behavioral confidence through personalized feedback. This creates a synergistic association between “technological empowerment” and “psychological adaptation.” Theoretically, our findings extend social cognitive theory into digital health contexts by revealing dynamic “environment–behavior–psychology” couplings. Practically, we recommend balancing data precision with psychological adaptability in technology design. Implementing tiered feedback systems can optimize health management experiences, establishing an actionable framework for digital interventions targeting HA among middle-aged individuals.

## 1 Introduction

In recent years, the rapid advancement of technology and paradigm shifts in health management concepts have led to the widespread adoption of sports technology devices (STDs) among middle-aged cohorts (MACs). These devices demonstrate substantial advantages in enhancing personal health monitoring capabilities and promoting healthy lifestyles while also playing a critical role in alleviating middle-aged health anxiety (HA). HA—a psychological state stemming from uncertainty about health status and perceived risks—has been empirically linked to emotional instability, diminished quality of life, and mental health deterioration in middle-aged individuals (1). Through wearable technologies, big data analytics, and intelligent feedback mechanisms, STDs not only enable real-time tracking of physical activity and health metrics but also employ data-driven approaches to strengthen users’ health control perception (HCP) and self-efficacy reconstruction (SER), thereby effectively buffering negative emotions associated with HA (2).

From a theoretical perspective, the psychological mechanisms underlying health behaviors can be understood as a sequence of self-regulatory processes. Bandura’s (3) self-efficacy theory posits that individuals’ confidence in their capabilities serves as a crucial determinant in initiating health behaviors. Building on this cognitive foundation, Ryan and Deci’s (4) self-determination theory emphasizes how such confidence can be sustained through intrinsic motivation, thereby supporting long-term behavioral maintenance. Furthermore, Lachman and Weaver (5) extend this framework by demonstrating that perceptions of control operate as a connecting mechanism between self-beliefs and concrete health decisions. Together, these theories form a coherent pathway from efficacy beliefs to motivated action, which is empirically supported by Vivien Hajak et al. (6), who found that exercise self-efficacy SER mediates the relationship between physical engagement and psychological improvement.

This study makes several key contributions. Primarily, it is the first to construct and test a chain mediation model integrating HCP and SER, examining their sequential role in the relationship between STD usage and HA among middle-aged individuals. This approach directly addresses a gap in the literature, which has traditionally examined health management, psychological adaptation, and technological applications in isolation. By empirically testing how these dimensions interact within a unified framework, our research provides a more nuanced theoretical understanding and offers concrete, mechanism-based guidance for designing health interventions for MACs.

Meanwhile, we developed an integrated theoretical framework. Using the “Environment–Cognition–Behavior” interaction proposed by social cognitive theory as the foundational pathway, we incorporated key insights from self-determination theory to explain how sports technology devices stimulate users’ intrinsic motivation for sustained engagement by satisfying their three fundamental psychological needs: autonomy, competence, and relatedness. At the same time, the health belief model was introduced to illustrate how such devices achieve rational calibration of health threats and intuitive visualization of management benefits through quantitative monitoring, thereby reshaping individuals’ sense of health control. This dual process of “motivation stimulation–cognition restructuring” jointly facilitates the reconstruction of self-efficacy, ultimately leading to the alleviation of health anxiety.

## 2 Literature review and hypotheses

### 2.1 Sports technology device usage and middle-aged health anxiety

The rapid evolution of exercise monitoring technologies has created new pathways for health management in MACs. Existing studies indicate that regular physical exercise significantly reduces anxiety levels—particularly in MACs—through physiological improvements and enhanced psychological regulation (7). By constructing digital exercise support systems, STDs effectively facilitate the establishment of structured exercise routines in MACs, thereby generating positive interventions for HA. For instance, Cadmus-Bertram et al.’s (8) randomized controlled trial with overweight/obese postmenopausal women demonstrated that self-monitoring via wearables (e.g., Fitbit) significantly increased participants’ daily activity levels, concurrently alleviating psychological stress and HA (8). Furthermore, STDs enhance exercise adherence to reinforce SER and mitigate anxiety (9) while creating supportive exercise environments through technological empowerment and social connectivity to improve psychological wellbeing (10). Advanced studies reveal that wearable STDs effectively reduce metabolic syndrome risks, indirectly lowering health threat perceptions (11), thereby providing robust theoretical support for STD-mediated anxiety reduction through exercise promotion.

Synthesizing these findings, STDs utilize real-time monitoring, data feedback, and personalized interventions to cultivate sustained exercise habits in MACs. We posit that this process alleviates excessive health concerns through key psychological mechanisms, primarily by enhancing perceived control and self-efficacy. Health anxiety often stems from a perception of uncertainty and helplessness regarding one’s health status. STDs’ real-time biofeedback transforms abstract health concerns into concrete, manageable data. This process of monitoring and mastering one’s physiological signals can significantly boost an individual’s perceived control over their health. Furthermore, as users follow personalized exercise regimens and achieve incremental goals, their confidence in managing their health through behavior is reinforced. Heightened self-efficacy and perceived control are well-established psychological antidotes to anxiety, as they reduce feelings of vulnerability and helplessness. Although studies specifically targeting MACs remain limited, existing evidence strongly supports STDs’ anxiety-reducing efficacy in this population. Consequently, based on the theoretical framework that STD usage enhances perceived control and self-efficacy, which, in turn, mitigates anxiety, we propose the following hypothesis:

*H1*: STD usage intensity will significantly negatively predict HA among middle-aged individuals, indicating that higher STD engagement corresponds to lower anxiety levels.

### 2.2 Mediating role of health control perception

Research on the psychological impacts of STD usage has increasingly focused on the mediating role of HCP between STD engagement intensity and HA among middle-aged individuals. Empirical evidence suggests that intensive STD usage significantly enhances individuals’ confidence and mastery in health management through real-time monitoring and feedback mechanisms, thereby elevating HCP (12). By enhancing SER—a critical psychological factor regulating behavioral and emotional responses—the approach effectively mitigates excessive health-related apprehensions (13).

Bandura’s health promotion model underscores the central role of “perceived self-efficacy”—the conceptual bedrock of HCP—in motivating health behaviors and improving mental wellbeing (14). The model posits that without belief in one’s capability to execute courses of action, sustained behavioral change is unlikely. McAuley and Blissmer’s study operationalized and validated this link, demonstrating that this very sense of efficacy regarding exercise is a strong predictor of subsequent physical activity (15). Therefore, these studies do not merely show correlations with HCP; they also theorize and empirically validate the construct of HCP itself, thereby providing a robust justification for STDs’ effect on anxiety via HCP enhancement.

Regarding HA, studies confirm that elevated HCP reduces anxiety stemming from health uncertainty and catastrophic interpretations of threats (16). Annesi et al.’s systematic review corroborates STDs’ positive association with health behavior promotion and emotional regulation (17). Collectively, these findings validate the theoretical model wherein STD usage intensity indirectly regulates HA among middle-aged individuals through HCP enhancement—a relationship intertwined with SER improvement and reflective of technology’s value in health management. Therefore, we hypothesize:

*H2*: STD usage intensity will significantly positively predict HCP.

*H3*: HCP will significantly negatively predict HA among middle-aged individuals.

*H4*: HCP will mediate the relationship between STD usage intensity and HA among middle-aged individuals.

### 2.3 Mediating role of self-efficacy reconstruction

The proliferation of STDs offers novel intervention pathways for MAC health management, potentially alleviating anxiety through SER enhancement. Research indicates that STDs’ real-time data feedback and personalized guidance significantly boost users’ health management confidence. For instance, feedforward control nursing combined with IKAP model health education increased self-monitoring participation among diabetes patients, effectively improving SER (18). Similarly, feedforward-controlled health interventions in post-thoracic surgery care demonstrated technology’s positive impact on SER by alleviating pain and improving quality of life (19).

Self-efficacy reconstruction’s anxiety-reducing effects are well documented across chronic disease studies. Experimental research demonstrated that enhancing SER in patients with hypertensive heart disease significantly reduced systolic/diastolic blood pressure and anxiety (20). In type 2 diabetes management, dietary guidance based on the health belief model modified disease-related attitudes and SER, indirectly alleviating glucose control-related stress (21).

Although direct evidence on SER’s mediating role between STD usage and MAC anxiety remains scarce, existing findings suggest that STDs may buffer HA through the technological empowerment of SER. Consequently, we propose:

*H5*: STD usage intensity will significantly positively predict SER.

*H6*: SER will significantly negatively predict HA among middle-aged individuals.

*H7*: SER will mediate the relationship between STD usage intensity and HA among middle-aged individuals.

### 2.4 Chain mediation effects of health control perception and self-efficacy reconstruction

Persistent STD usage enhances users’ health data monitoring capacity, thereby strengthening HCP (22). For example, WeChat-based general practice health management significantly improved SER and glycemic control in diabetes patients (23). As a belief in health management competence, HCP is intrinsically linked to SER. Studies reveal positive correlations between an internal health locus of control and SER in heart failure patients (24), while diabetes research demonstrates SER’s regulatory role in treatment adherence through health control beliefs (25).

Interventions combining health education and gratitude practices significantly increased HCP and SER while reducing anxiety in older hypertensive patients (26), further validating their synergistic relationship. SER’s anxiety-reducing effects are corroborated by Ding Weiguang et al., whose study demonstrated reductions in glycemic-related anxiety in type 2 diabetes patients through SER enhancement (27). Although chain mediation mechanisms involving HCP and SER in STDs’ anxiety-buffering effects remain underexplored, evidence from e-health literacy studies—where SER and treatment adherence sequentially mediated blood pressure reduction—supports this pathway’s plausibility. Accordingly, we hypothesize:

*H8*: HCP will significantly positively predict SER.

*H9*: HCP and SER will jointly mediate the relationship between STD usage intensity and HA among middle-aged individuals through a chain mediation mechanism.

### 2.5 Research model

**Figure 1:**
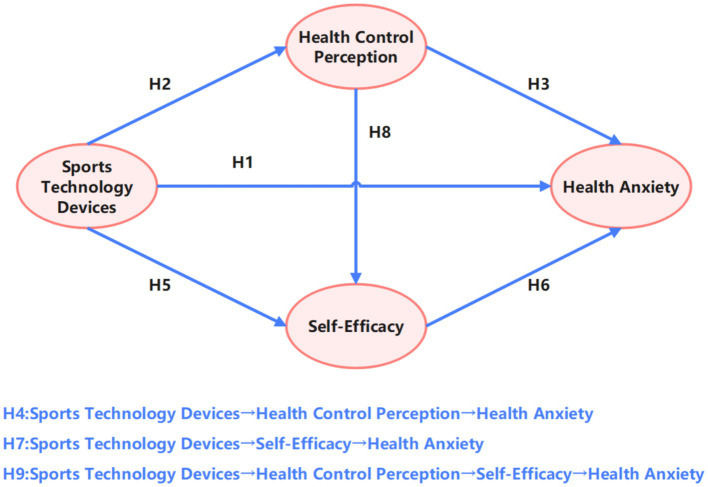
Research hypothesis model.

Based on the theoretical framework and empirical evidence presented, this study proposed the research hypotheses (Table 1). Meanwhile, this study established a chain mediation model (Figure 1) illustrating the relationships between sports technology device (STD) usage intensity, HA among middle-aged individuals (MHA), HCP, and SER. The conceptual model specifies the following:

**Table 1:** Research hypotheses.

Number	Hypotheses
H1	STD usage intensity will significantly negatively predict HA among middle-aged individuals, indicating that higher STD engagement corresponds to lower anxiety levels.
H2	STD usage intensity will significantly positively predict HCP.
H3	HCP will significantly negatively predict HA among middle-aged individuals.
H4	HCP will mediate the relationship between STD usage intensity and HA among middle-aged individuals.
H5	STD usage intensity will significantly positively predict SER.
H6	SER will significantly negatively predict HA among middle-aged individuals.
H7	SER will mediate the relationship between STD usage intensity and HA among middle-aged individuals.
H8	HCP will significantly positively predict SER.
H9	HCP and SER will jointly mediate the relationship between STD usage intensity and HA among middle-aged individuals through a chain mediation mechanism.

## 3 Research methods

### 3.1 Participants

This study used a stratified random sampling method across five provinces and 10 cities in Eastern, Central, and Western China to examine the chain mediation effect of STD usage on HA among middle-aged individuals through HCP and SER, targeting individuals aged 45–59 years. A total of 1,000 paper-based questionnaires were distributed, and 930 valid responses were retained after a systematic screening process that excluded 70 questionnaires (7.0%) based on the following criteria: missing values exceeding 10%, patterned responses, completion time under 5 min, and age outside the specified range. The resulting validity rate of 93.0% ensured that the sample met the 10:1 subject-to-item ratio required for structural equation modeling. In addition, the combined use of quantitative and logical verification minimized measurement error and provided a reliable basis for subsequent chain mediation analysis.

As presented in Table 2, the sample exhibited balanced demographic characteristics. Age distribution was as follows: 45–49 years (31.6%, *n* = 294), 50–54 years (35.4%, *n* = 329), and 55–59 years (33.0%, *n* = 307), with slightly higher representation in the 50–54 age group. The mean age was 51 years. Sex distribution showed that female participants slightly outnumbered male participants (52.2%, *n* = 485 vs. 47.8%, *n* = 445). Monthly income was distributed across four tiers: <¥2,000 (24.9%, *n* = 232), ¥2,000–3,000 (25.4%, *n* = 236), ¥3,000–4,000 (25.4%, *n* = 236), and >¥4,000 (24.3%, *n* = 226), showing no significant concentration. The mean income was 2,254 yuan. Residence was nearly equally represented, with urban participants accounting for 52.5% (*n* = 488) and rural participants 47.5% (*n* = 442). Geographic distribution included Eastern (33.1%, *n* = 308), Central (32.5%, *n* = 302), and Western (34.4%, *n* = 320) regions, ensuring spatial representativeness.

**Table 2:** Demographic characteristics of participants (*N* = 930).

Var.	Category	Frequency	Percentage (%)
Age	45 ~ 49	294	31.6
	51 ~ 55	329	35.4
	55 ~ 59	307	33.0
Sex	Male	445	47.8
	Female	485	52.2
Monthly Income	<¥2,000	232	24.9
	2,000 ~ 3,000	236	25.4
	3,000 ~ 4,000	236	25.4
	>¥4,000	226	24.3
Residence	Urban	488	52.5
	Rural	442	47.5
Geographic Region	Eastern China	308	33.1
	Central China	302	32.5
	Western China	320	34.4
	Total	930	100

The sample’s balanced distribution across age, sex, income, residence, and geographic region aligns with social science research standards, thereby supporting the validity of the subsequent analyses. Nevertheless, the generalizability of the findings is constrained by regional and sampling limitations.

### 3.2 Measurement instruments

#### 3.2.1 Sports technology usage intensity scale

The “Survey Questionnaire on the Intensity of Use of Sports Technology Products” (28) employed in this study is a self-designed instrument that has not been published. Developed through the integration of established domestic and international scales with the unified theory of acceptance and use of technology (UTAUT) model (28) and STD application contexts, this scale defines three core dimensions: Behavioral Frequency: Regularity of STD engagement; Functional Depth: Complexity and proactivity in feature utilization; and Continuance Dependency: Willingness to reuse after interruption and psychological reliance.

A six-member expert panel (psychology and sports science scholars) conducted scientific validation of the dimensions and items using the Delphi method. A total of eight middle-aged participants (four male and four female) subsequently assessed the questionnaire’s readability, leading to semantic refinement of ambiguous items. Following iterative revisions (merging redundant items and eliminating irrelevant content), a second round of expert consultation confirmed that all item coefficients of variation (CV) were < 0.25, establishing the preliminary scale.

To ensure psychometric robustness, a pilot study was conducted with 80 participants recruited from supermarkets in Jining, Shandong Province (76 valid responses; 95% response rate). Cross-validation analysis using SPSS 26.0 demonstrated the following:

All factor loadings > 0.45; KMO = 0.796, Bartlett’s test: χ^2^ = 305.757 (*p* < 0.001); Cronbach’s *α* = 0.806.

The final scale comprises eight items across the three dimensions and is rated on a 5-point Likert scale (total score range: 8–40; higher scores indicate greater STD engagement intensity). In the main study, psychometric properties remained robust: KMO = 0.881, Bartlett’s test: *χ*^2^ = 1341.666 (*p* < 0.001); Cronbach’s *α* = 0.779; χ^2^/df = 1.008; CFI = 0.996; TLI = 0.998; RMSEA = 0.003 (Table 3).

**Table 3:** Reliability test results.

Dimension	No.	Item
Behavioral Frequency	Q1	I typically use sports technology devices (STDs; e.g., wearables, apps) for [X] days per week.
Functional Depth	Q2	I regularly review health reports generated by STDs (e.g., weekly/monthly summaries).
	Q3	I adjust exercise intensity based on STD-monitored data (e.g., elevated heart rate alerts).
	Q4	I utilize personalized training plans provided by STDs (e.g., recommended exercise courses).
	Q5	I employ STD social features (e.g., activity check-ins, data sharing) to motivate regular exercise.
Continuance Dependency	Q6	I perceive my exercise regimen as incomplete when I forget to use STDs.
	Q7	I continue using STDs despite short-term failure to achieve expected outcomes.
	Q8	I consider STDs fundamentally important for my health management.

#### 3.2.2 HA inventory

The short-form HA Inventory (HAI), originally developed by Salkovskis (29) and cross-culturally adapted by Yuqun Zhang et al. (30), assesses two dimensions: (1) concerns about illness likelihood and (2) fear of negative illness consequences. This 18-item instrument employs a 5-point Likert scale. In this study, psychometric analysis revealed excellent validity (KMO = 0.961; Bartlett’s χ^2^ = 4187.662, *p* < 0.001) and reliability (Cronbach’s *α* = 0.887).

#### 3.2.3 Multidimensional health locus of control scale

The Multidimensional Health Locus of Control Scale (MHLC) (31) evaluates three dimensions: internal locus (6 items), powerful others (6 items), and chance (6 items), for a total of 18 items rated on a 5-point Likert scale. Subscale scores range from 6 to 30, and total scores range from 6 to 90; higher scores indicate a stronger orientation toward the respective dimension. The scale demonstrated strong validity (KMO = 0.961; Bartlett’s χ^2^ = 4094.937, *p* < 0.001) and reliability (Cronbach’s *α* = 0.887).

#### 3.2.4 General self-efficacy scale

SER was measured using the General Self-Efficacy Scale validated by Luo and Xie (32). This 10-item instrument, scored on a 5-point Likert scale (total range: 10–50; higher scores indicate stronger SER), exhibited robust psychometric properties: KMO = 0.899, Bartlett’s χ^2^ = 1606.507 (*p* < 0.001), and Cronbach’s *α* = 0.793.

### 3.3 Data processing and analysis

Statistical analyses were conducted using SPSS 27.0 and PROCESS 4.0, following these steps: Data preparation: Classification, transformation, and computation of valid data; common method bias (CMB) control: Harman’s single-factor test for CMB assessment; and psychometric validation: Cronbach’s α for reliability and Bartlett’s test for validity.

Hypothesis testing involved correlation and linear regression analyses to examine relationships among variables. Bootstrap resampling (5,000 iterations) was used to assess both simple and chain mediation effects of STD usage intensity, HCP, and SER on HA.

## 4 Research results

### 4.1 Common method bias test

Exploratory factor analysis (EFA) using Harman’s single-factor test showed that the first unrotated principal component explained 27.02% of variance (<40% threshold), indicating no severe CMB. However, given the limited sensitivity of Harman’s test, we further employed the more rigorous “unmeasured latent method factor” approach for validation. This method directly estimates the extent of common method bias (CMB) by introducing a latent method factor into the structural equation model. Using the AMOS software, we constructed two models: (1) a baseline model containing only the four substantive variables (STD, HA, HCP, and SER) and (2) an extended model incorporating a common method factor (CMF) that loaded on all measurement items, with its path coefficients constrained to be equal. Model comparison revealed that the inclusion of the CMF did not significantly improve model fit (ΔCFI = 0.012, ΔTLI = 0.011, ΔRMSEA = −0.001), and the CMF accounted for only 8.7% of the total variance—well below the recommended threshold of 25%. These results strongly suggest that the relationships between variables in this study were not substantially distorted by common method bias. Additional precautions included the following: Anonymous responses, separation of independent and dependent variable items, and use of multi-source scales with clear phrasing.

### 4.2 Correlation analysis

Descriptive statistics and Pearson correlations are presented in Table 4. The key findings included the following: STD usage intensity was negatively correlated with HA (*r* = −0.817, *p < 0.01), STD usage was positively correlated with HCP (*r* = 0.700, *p < 0.01) and SER (*r* = 0.567, *p < 0.01), HA was negatively correlated with HCP (*r* = −0.715) and SER (*r* = −0.624), and HCP and SER were significantly positively correlated (*r* = 0.578, *p < 0.01).

**Table 4:** Means, standard deviations, and correlations among key variables.

Var.	*M*	SD	STD	HA	HCP	SER
STD	2.99	0.75				
HA	3.00	0.70	−0.817**			
HCP	3.00	0.69	0.700**	−0.715**		
SER	2.99	0.71	0.567**	−0.624**	0.578**	

*p* < 0.05; *p < 0.01; **p < 0.001 (consistent across analyses).

Since the correlation of some variables was relatively high, we conducted a collinearity diagnosis. The VIF values for STD, HCP, and SER were 2.127, 2.168, and 1.628, respectively. There was no significant collinearity among the variables.

Univariate linear regression analyses were conducted to examine the individual predictive effects of sports technology device (STD) usage intensity, HCP, and SER on HA among middle-aged individuals (Table 5). Key parameters—including the regression coefficient (B), standard error (SE), standardized coefficient (*β*), *T*-value, *F*-value, coefficient of determination (*R*^2^), and adjusted *R*^2^ (R^2^adj)—are systematically reported.

**Table 5:** Univariate regression analysis of the effects of STD usage intensity, HCP, and SER on HA among middle-aged individuals.

Var.	MAC HA						
	*B*	SE	*β*	*T*	*F*	*R* ^2^	R^2^adj
Const.	5.279	0.055					
STD	−0.764	0.018	−0.817	−43.109	1858.343	0.667	0.667
Const.	5.161	0.071					
HCP	−0.721	0.023	−0.715	−31.195	973.154	0.512	0.511
Const.	4.827	0.077					
SER	−0.613	0.025	−0.624	−24.301	590.523	0.389	0.388

For STD usage intensity, the regression model demonstrated the following results: *B* = −0.764 (SE = 0.018), *β* = −0.817, *T*(928) = −43.109; *F*(1, 928) = 1858.343***; *R*^2^ = 0.667 (R^2^adj = 0.667).

This indicates that STD usage intensity accounted for 66.7% of the HA variance, exhibiting the strongest negative predictive effect.

For HCP, the model showed the following results: *B* = −0.721 (SE = 0.023), *β* = −0.715, *T*(928) = −31.195; *F*(1, 928) = 973.154***; *R*^2^ = 0.512 (R^2^adj = 0.511). HCP accounted for 51.2% of the variance and exhibited the second-largest effect.

For SER, the model yielded the following results: *B* = −0.613 (SE = 0.025), *β* = −0.624, *T*(928) = −24.301; *F*(1, 928) = 590.523***; *R*^2^ = 0.389 (R^2^adj = 0.388).

Notably, all intercepts were significant (*B* = 5.279–4.827), reflecting elevated baseline anxiety levels when predictors were null. Standardized coefficients were ranked as follows: STD usage intensity (|*β*| = 0.817) > HCP (|*β*| = 0.715) > SER (|*β*| = 0.624). The minimal discrepancy between *R*^2^ and R^2^adj across models (*Δ* ≤ 0.001) confirmed a robust model fit without overfitting.

### 4.3 Mediation effect tests

Model 6 in PROCESS was conducted. Bootstrap-mediated path analysis (5,000 resamples) revealed a significant mediation association (Table 6). The total effect of sports technology device (STD) usage intensity on HA among middle-aged individuals was −0.7646 (SE = 0.0200, 95% CI [−0.7995, −0.7297]). The direct effect accounted for 68.88% of the total effect (B = −0.5267, SE = 0.0235, 95% CI [−0.5729, −0.4806]), while the total indirect effect explained 31.12% of the total effect (B = −0.2379, SE = 0.0194, 95% CI [−0.2770, −0.2005]). These results indicate that, although the direct effect predominated, the mediation pathways collectively contributed substantial explanatory power.

**Table 6:** Mediation pathway analysis using bootstrap testing.

Effect	Path	Effect size	Standard error	LLCL	ULCL	Percentage of the total effect
Total effect	Direct Path	−0.7646	0.0200	−0.7995	−0.7297	100
Direct effect		−0.5267	0.0235	−0.5729	−0.4806	68.88
Total indirect effect		−0.2379	0.0194	−0.2770	−0.2005	31.12
Indirect effect	Pathway 1	−0.1438	0.0165	−0.1773	−0.1116	18.81
	Pathway 2	−0.0525	0.0095	−0.0728	−0.0357	6.87
	Pathway 3	−0.0416	0.0068	−0.0558	−0.0294	5.44

Further Analysis of Mediation Pathways. Detailed examination of the three mediation pathways revealed distinct effect magnitudes (Table 6): Pathway 1 (STD → HCP → SER → Anxiety): Effect = −0.1438 (SE = 0.0165, 95% CI [−0.1773, −0.1116]), contributing 18.81% of the total effect (largest mediation effect); Pathway 2 (STD → HCP → Anxiety): Effect = −0.0525 (SE = 0.0095, 95% CI [−0.0728, −0.0357]), accounting for 6.87% of the total effect; and Pathway 3 (STD → SER → Anxiety): Effect = −0.0416 (SE = 0.0068, 95% CI [−0.0558, −0.0294]), explaining 5.44% of the total effect.

**Figure 2:**
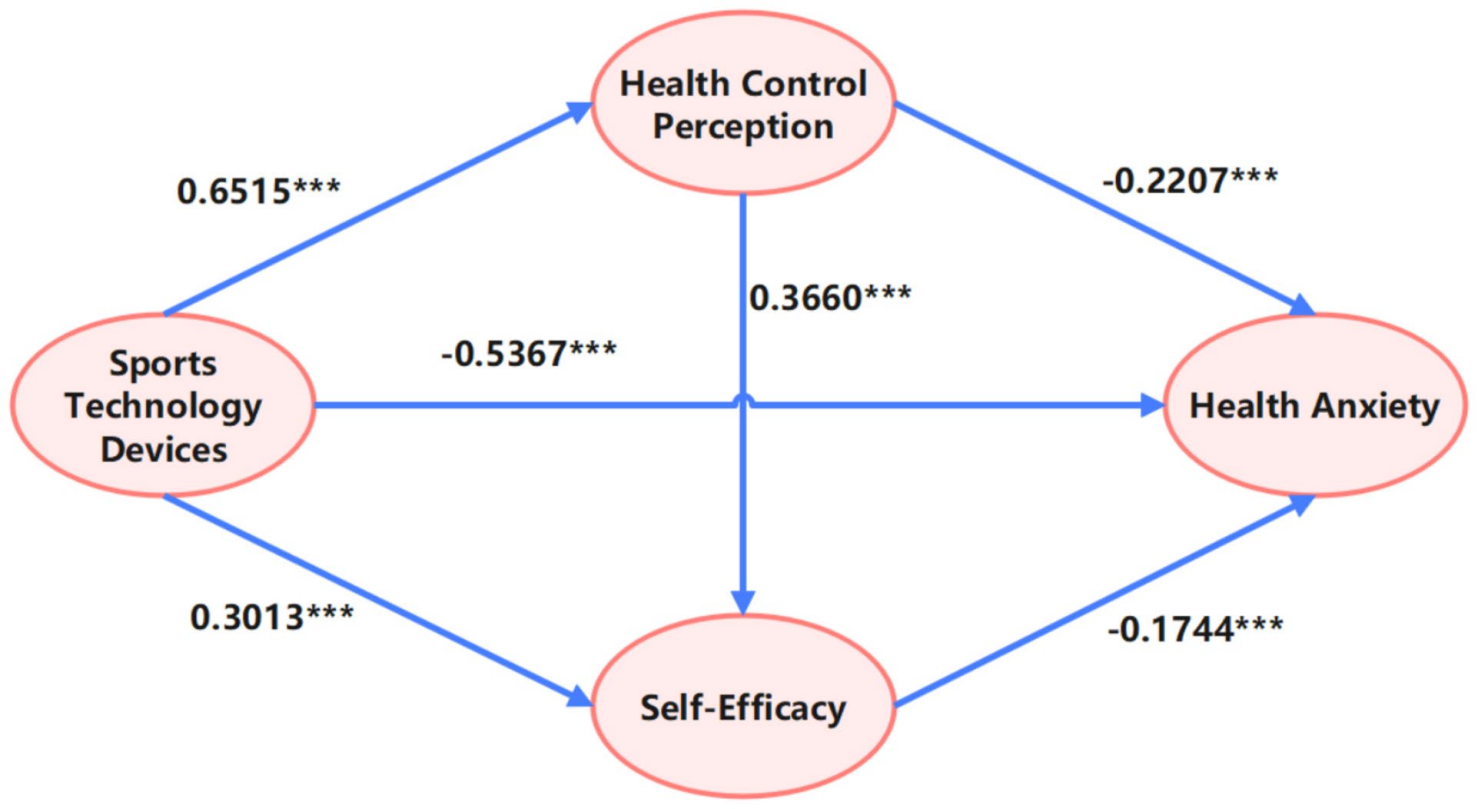
Chain mediation model of the effect of STD usage on HA among middle-aged individuals (MHA).

The effect sizes were statistically non-zero (see Figure 2) (Pathway 1 > 2 > 3), with all CIs excluding zero, confirming their significance. Model integrity was validated by the additive consistency among the total effect (−0.7646), direct effect (−0.5267), and total indirect (−0.2379), with negligible residual error (*Δ* < 0.0001), supporting the model’s structural soundness. These findings collectively affirm the statistical validity of the mediation relationship, with Pathway 1 identified as the core mediating route.

## 5 Discussion

### 5.1 Sports technology device usage significantly reduces middle-aged health anxiety

The pervasive rise of health anxiety (HA) in modern society is particularly pronounced among middle-aged cohorts (MACs) due to physiological decline and multi-role stressors. Smart technology devices (STDs) offer novel strategies for mitigating such anxiety, primarily through real-time physiological monitoring and data feedback, which enhance objective health awareness and reduce uncertainty-driven anxiety (33). This relationship aligns with cognitive-behavioral theory, where technology-assisted interventions facilitate behavioral activation and the cognitive restructuring of maladaptive health beliefs (34).

Notably, STD efficacy is moderated by health literacy. High-literacy users are better able to interpret device data rationally, thereby avoiding misinterpretation-induced panic (35), while leveraging actionable insights to transform STDs into effective health management tools (36). Furthermore, technology-enabled social support systems—such as online health communities and social features embedded within STDs—strengthen adherence to health behaviors and resonate with MAC’s need for belongingness (37–39).

However, overreliance on monitoring may foster “data obsession” or cyberchondria, as abnormal biometric alerts can trigger transient anxiety (40, 41). Therefore, STD design must balance transparency with psychological adaptability, for example, through algorithmic filtration of non-critical alerts or the integration of embedded mindfulness modules.

The health belief model offers a complementary theoretical perspective, framing STDs as tools that enhance perceived threat awareness (e.g., via metabolic trend charts) while reinforcing behavioral benefits through visualized goal-achievement feedback. This creates a closed-loop system of “risk alert–behavioral reinforcement–positive feedback,” synergizing with cognitive restructuring and self-efficacy reinforcement mechanisms.

Future research should explore personalized recommendation systems and address ethical concerns such as data privacy (41, 42). Ultimately, STDs function as pivotal mediators in reconstructing MACs’ health narratives through “technology–behavior–cognition” linkages, providing innovative pathways for anxiety mitigation in the digital era.

### 5.2 Mediating role of health control perception

The proliferation of STDs has not only provided MACs with accessible health management tools but also demonstrated significant potential in alleviating HA. This study posits that STD usage enhances individuals’ HCP through real-time data monitoring, health assessments, and behavioral feedback, serving as a critical psychological mechanism for mitigating HA among middle-aged individuals.

Theoretically, HCP functions as a psychological resource that strengthens self-efficacy when confronting health threats, thereby reducing negative emotional responses (43). Furthermore, STDs improve health information transparency and operationalizability, fostering adaptive cognitive patterns that empower users to adopt proactive coping strategies against health uncertainties—a process that reinforces HCP and mitigates age-related anxiety (44).

From a neurobiological perspective, the formation of HCP is supported by research on self-efficacy and perceived control. Studies have shown that a heightened sense of control is associated with increased activation in the prefrontal cortex (PFC) and reduced amygdala reactivity, which are key regions for cognitive regulation and fear processing. The repeated positive feedback and successful health management experiences provided by STDs may therefore facilitate neuroplasticity in these circuits, strengthening top-down control over threat responses (45). Consequently, enhanced HCP likely contributes to a multi-layered emotional regulation system, not only by directly modulating neural circuits of anxiety but also indirectly by promoting healthier lifestyles and social engagement, thereby improving MACs’ adaptability to life transitions (46).

In conclusion, STDs alleviate HA among middle-aged individuals through HCP mediation by directly improving cognitive-emotional regulation and indirectly optimizing health behaviors. This dual-pathway model provides a foundational framework for designing targeted, technology-mediated interventions to mitigate MAC HA.

### 5.3 Mediating role of self-efficacy reconstruction

This study elucidates the complex relationship linking sports technology device (STD) usage to HA among middle-aged individuals through SER mediation. Empirical evidence suggests that MACs, facing physiological decline and multi-role stressors, are particularly vulnerable to HA cycles (47). STDs—including wearables and health management apps—not only provide real-time biometric monitoring and personalized exercise guidance but also enhance users’ health management efficacy via behavioral feedback mechanisms (48).

The SER process emerges as a critical pathway for anxiety mitigation. Positive feedback from STDs (e.g., step goal achievements, stable heart rate reports) reinforces SER, thereby reducing anxiety triggered by health uncertainties. These findings align with Chen et al.’s (49) conclusions that exercise-induced SER alleviates occupational burnout, suggesting cross-contextual links between technology-enhanced physical practices and psychological adaptation.

Crucially, SER reconstruction is contextually embedded. While prior studies emphasize the role of perceived ease of use and information quality in health information-seeking behaviors (50), this research extends these insights by identifying STD technical features (e.g., intuitive interfaces, data visualization) as indirect anxiety regulators through the enhancement of operational confidence. For instance, clear visualization of exercise outcomes and health trends enables objective risk appraisal, mitigating anxiety arising from information overload (48). Furthermore, MACs’ chronic disease concerns (51) are addressed through STDs’ longitudinal tracking and risk monitoring, fostering a “preventive health management” cognitive framework underpinned by cumulative SER development (52).

However, SER mediation efficacy is moderated by individual differences and technology compatibility. Wang et al. (53) highlighted the influence of intergenerational support and digital literacy on health behaviors, while this study shows that technology aversion among MACs may exacerbate anxiety due to operational barriers—consistent with Stone et al.’s (54) finding of positive SER–anxiety correlations in low-efficacy groups. Future STD designs should balance usability with user education, employing tiered functionalities to accommodate diverse efficacy levels. A key limitation of this study is the underexploration of social support’s moderating role (e.g., peer interactions, familial health norms). Theoretically, this study integrates technological, psychological, and behavioral dimensions into a unified framework, providing SER-based insights for digital health interventions.

### 5.4 Chain mediation effects of health control perception and self-efficacy reconstruction

The chain mediation relationship involving HCP and SER carries significant theoretical and practical implications. STDs enhance HCP through real-time data monitoring and feedback (55), which directly informs health-related decisions and indirectly reshapes psychological states via SER reinforcement. For example, monitoring incremental progress toward fitness goals via wearables (e.g., heart rate monitors, activity trackers) reinforces users’ confidence in health self-management (56). Elevated HCP motivates proactive health behaviors (57), whose cumulative outcomes further consolidate SER (58).

MACs’ susceptibility to HA—amplified by physiological aging and social role conflicts (59, 60)—may be mitigated through this pathway. Hypertension studies confirm positive HCP–SER correlations and their joint influence on clinical outcomes (61), supporting STDs’ applicability in chronic disease management. However, balancing exogenous HCP (e.g., device dependency) and endogenous HCP (e.g., self-trust) remains critical. Over-reliance on external data may undermine intrinsic control beliefs, jeopardizing long-term health behavior sustainability (62, 63). Therefore, future designs should harmonize technological empowerment with psychological autonomy to prevent technology-induced disempowerment (64, 65).

From a social cognitive perspective, HCP–SER interactions exemplify Bandura’s triadic reciprocity among personal, behavioral, and environmental factors, with STD efficacy modulated by individual cognition and technology acceptance (66). Techno-skeptic users may experience heightened anxiety due to data interpretation difficulties, whereas high-digital-literacy groups derive greater benefits (67). These findings not only delineate STDs’ mental health pathways but also inform personalized intervention strategies. Future research should explore this chain mechanism’s cross-cultural stability and boundary conditions.

### 5.5 The potential risks of excessive use or reliance on digital health technology data

Although digital health technologies demonstrate significant potential for alleviating middle-aged health anxiety, their widespread application necessitates vigilance against potential risks arising from data overuse and technological dependency. Research indicates that users may enter a state of “compulsive data monitoring” due to excessive focus on quantified health metrics, leading to irrational reliance on device-generated data, such as heart rate and step counts. Even normal physiological fluctuations may be misinterpreted as health threats, ultimately exacerbating anxiety. This phenomenon, defined as “quantified self-burnout,” fundamentally stems from an imbalance between technology use and health cognition (68). Concurrently, technological dependency may erode users’ intrinsic sense of health control. When health decisions rely excessively on algorithmic feedback, individuals may neglect bodily intuition, causing self-efficacy to shift from “proactive management” to “passive compliance” (69).

Furthermore, centralized health data platforms carry the risk of data leakage, and user concerns about data misuse may reduce technological engagement, particularly among middle-aged cohorts (70). A more insidious risk arises from inherent algorithmic biases—for instance, “health thresholds” based on standardized populations may fail to account for individuals with chronic conditions or unique physiological characteristics, thereby exacerbating health inequities. Studies reveal that medical digital technologies may systematically underestimate health risks for minority groups due to insufficient data representation, further marginalizing vulnerable users (71). Clearly, future technological designs must integrate “explainable artificial intelligence” to enhance transparency in data logic, helping users comprehend metric significance, while developing tiered feedback mechanisms to filter non-critical alerts and reduce information overload. In addition, user education programs should be strengthened to cultivate critical health data literacy, transforming technology from a “substitutive tool” to an “empowering partner.” Only through the refinement of ethical frameworks and interdisciplinary collaboration can a dynamic equilibrium between digital health innovation and psychosocial adaptation be achieved.

## 6 Conclusion and future directions

### 6.1 Conclusion

This study confirmed that STD usage intensity significantly negatively predicted HA among middle-aged individuals through a chain mediation relationship involving HCP and SER. Empirical findings demonstrated that technology-enabled real-time monitoring and feedback enhanced individuals’ objective health awareness and proactive management capabilities, thereby strengthening HCP. Subsequent positive behavioral feedback (e.g., achieving exercise goals, improving physiological metrics) reinforced users’ confidence in SER, ultimately forming a progressive buffering chain: technological intervention → control enhancement → efficacy accumulation → anxiety mitigation. These results align with the “environment-behavior-psychology” triadic reciprocity principle in social cognitive theory, revealing the intrinsic logic of how digital tools reshape health behaviors through psychological mechanisms.

### 6.2 Research limitations

The current study has several limitations that warrant consideration. (1) The cross-sectional design inherently restricts the ability to establish causal relationships among the variables, as temporal precedence and longitudinal dynamics cannot be confirmed. While the findings suggest associations between STD usage, HCP, SER, and reduced HA, causality remains speculative without experimental or time-lagged data. (2) The reliance on self-reported measures introduces potential biases, such as social desirability or recall inaccuracies, particularly regarding HA and technology usage patterns. (3) Despite efforts to ensure geographic diversity within China, the sample’s cultural homogeneity limits the generalizability of the findings to populations with different sociocultural contexts, healthcare systems, or technological adoption patterns. (4) The study’s exclusive focus on MACs (45–59 years) may overlook age-specific variations in technology acceptance and psychological mechanisms, limiting insights into younger or older demographics. (5) The self-designed Sports Technology Usage Intensity Scale, although validated, lacks established comparability with standardized instruments, potentially affecting the interpretation of the results. (6) The model did not account for confounding factors such as pre-existing mental health conditions, baseline physical health status, or access to healthcare resources, which might influence both STD usage and anxiety outcomes. (7) The absence of longitudinal follow-up precludes an assessment of the sustainability of the observed association, leaving questions about whether the buffering mechanisms persist over time or diminish with prolonged device use. Addressing these limitations in future research would strengthen the validity and applicability of the proposed theoretical framework. (8) The study may also have unmeasured or omitted variables that simultaneously affect device usage tendencies and anxiety levels.

### 6.3 Future directions

#### 6.3.1 Theoretical implications

While the chain mediation model grounded in social cognitive theory provides an initial framework for understanding technology-psychology interactions, its theoretical depth and boundaries require further exploration. Future research should:

Integrate self-determination theory and health belief model to clarify the dynamic balance between intrinsic motivation (e.g., autonomy needs) and extrinsic technological incentives (e.g., data feedback) in sustaining health behaviors.

Develop longitudinal models to examine the temporal evolution patterns of HCP and SER across STD usage phases (adaptation, maintenance, and burnout) and their differential impacts on anxiety.

Explore neurobiological mechanisms using fMRI or fNIRS to identify neural correlates (e.g., prefrontal-striatal connectivity) underlying technology-enhanced HCP/SER.

Investigate cultural specificity by comparing collectivist contexts (e.g., familial support synergies) and individualist contexts (e.g., autonomy-technology conflicts) to refine cross-cultural digital health theories.

#### 6.3.2 Practical implications

To optimize STD applications in middle-aged health management, it is necessary to: Design cognitive-adaptive interfaces that dynamically adjust data presentation (e.g., trend graphs for low-literacy users) and provide contextual interpretations (e.g., “Elevated heart rate may reflect exercise intensity; adjust breathing rhythm”).

Implement tiered feedback systems with incremental micro-goals (e.g., phased step targets: 5,000 → 8,000 daily steps) and embedded mindfulness modules (e.g., post-exercise breathing guidance) to reinforce SER and alleviate monitoring-induced stress.

Foster technology-social support integration through family health data sharing or community challenges to leverage social comparison and peer motivation.

Prioritize ethical design through “selective transparency” data privacy controls and digital literacy workshops to mitigate anxiety risks among technology-averse populations.

## Data Availability

The original contributions presented in the study are included in the article/Supplementary material, further inquiries can be directed to the corresponding author/s.

## References

[1] Alsenany SA. Psychological predictors of gerascophobia among middle-aged and older adults: the role of health anxiety and body image satisfaction. Geroscience. (2025):1–13. doi: 10.1007/S11357-025-01807-2 40711671 PMC12972265

[2] Qiao Y. Key technologies and application frontiers of sports smart wearable devices. J Capital Ins Physical Educ. (2025) 37:364–74. doi: 10.14036/j.cnki.cn11-4513.2025.04.002

[3] Bandura A. Self-efficacy mechanism in human agency. Am Psychol. (1982) 37:122–47. doi: 10.1037/0003-066X.37.2.122

[4] Ryan RM Deci EL. Self-determination theory and the facilitation of intrinsic motivation, social development, and well-being. Am Psychol. (2000) 55:68–78. doi: 10.1037/0003-066X.55.1.68, 11392867

[5] Lachman ME Weaver SL. The sense of control as a moderator of social class differences in health and well-being. J Pers Soc Psychol. (1998) 74:763–73. doi: 10.1037/0022-3514.74.3.763, 9523418

[6] Hajak V Grimm S Gruszczyńska E Kroemeke A Józefacka N Warner LM. Experimental paradigm to test the effects of providing social support: study protocol of the PROSPECT trial (study 2). BMC Psychol. (2025) 13:74. doi: 10.1186/s40359-024-02319-y, 39871367 PMC11773939

[7] Asmundson GJ Fetzner MG Deboer LB Powers MB Otto MW Smits JA. Let's get physical: A contemporary review of the anxiolytic effects of exercise for anxiety and its disorders. Depress Anxiety. (2013) 30:362–73. doi: 10.1002/da.22043, 23300122

[8] Cadmus-Bertram L Marcus BH Patterson RE Parker BA Morey BL. Use of the Fitbit to measure adherence to a physical activity intervention among overweight or obese, postmenopausal women: self-monitoring trajectory during 16 weeks. JMIR Mhealth Uhealth. (2015) 3:e96. doi: 10.2196/mhealth.4229, 26586418 PMC4705008

[9] Lai X Bo L Wu Z Chen B Zhu H Chen L . Effects of wearable device-guided lower limb resistance exercise on pre-frail older adults. Beijing Med J. (2021) 43:1138–41. doi: 10.15932/j.0253-9713.2021.11.026

[10] Zhang Y Li H. Ecosystem analysis of adolescents' participation in physical exercise. J Liaoning Radio TV Univ. (2023) 1:75–8. doi: 10.19469/j.cnki.1003-3297.2023.01.0075

[11] Joung IK An HS Bang JS Kim KJ. Comparative effectiveness of wearable devices and built-in step counters in reducing metabolic syndrome risk in South Korea: population-based cohort study. JMIR Mhealth Uhealth. (2025) 13:e64527. doi: 10.2196/64527 39999338 PMC11878715

[12] Mercer K Li M Giangregorio L Burns C Grindrod K. Behavior change techniques present in wearable activity trackers: a critical analysis. JMIR Mhealth Uhealth. (2016) 4:e40. doi: 10.2196/mhealth.4461, 27122452 PMC4917727

[13] Luszczynska A Scholz U Schwarzer R. The general self-efficacy scale: multicultural validation studies. J Psychol. (2005) 139:439–57. doi: 10.3200/JRLP.139.5.439-457, 16285214

[14] Bandura A. Health promotion by social cognitive means. Health Educ Behav. (2004) 31:143–64. doi: 10.1177/1090198104263660, 15090118

[15] McAuley E Jerome GJ Elavsky S Marquez DX Ramsey SN. Predicting long-term maintenance of physical activity in older adults. Prev Med. (2003) 37:110–8. doi: 10.1016/S0091-7435(03)00089-6, 12855210

[16] Affrunti NW Geronimi EM Woodruff-Borden J. Language of perfectionistic parents predicting child anxiety diagnostic status. J Anxiety Disord. (2015) 30:94–102. doi: 10.1016/j.janxdis.2015.01.001, 25618460

[17] Annesi JJ. Relations of change in fruit and vegetable intake with overall energy reduction and physical activity with weight change: assessing theory-based psychosocial mediators. J Sport Health Sci. (2019) 8:394–9. doi: 10.1016/j.jshs.2018.08.005, 31333894 PMC6620207

[18] Zhong L. Effects of feedforward control nursing combined with IKAP model health education on diabetes self-efficacy. Heilongjiang J Tradit Chin Med. (2024) 53:160–2.

[19] Li Z Li N. Application of feedforward control-based health education in post-thoracic surgery continuing care. Chin Health Care. (2024) 42:96–8.

[20] Huang Y. Effects of staged nursing based on self-transcendence theory on health literacy, self-efficacy, and blood pressure control in hypertensive heart disease patients. Cardiovasc Disease Prevent Treat Knowledge. (2024) 14:90–3.

[21] Zhu Y Wang X Yan S. Application of dietary guidance under the health belief model in blood glucose control for type 2 diabetes patients. Qilu Nurs J. (2023) 29:133–6.

[22] Tan Y Liang J Zhang H. Impact of health guidance on blood glucose control and self-efficacy in community type 2 diabetes patients. Shenzhen J Integr Tradit Chin West Med. (2021) 31:195–6. doi: 10.16458/j.cnki.1007-0893.2021.12.087

[23] Wu J Chen Y Huang M Liu K. Effectiveness of WeChat platform-based general practice health management model in controlling type 2 diabetes. China Med Pharm. (2024) 14:175–9. doi: 10.20116/j.issn2095-0616.2024.01.41

[24] Leo LE Simon M Grothe KB Boudreaux E Bodenlos JS Wallston K . The interaction of locus of control, self-efficacy, and outcome expectancy in relation to HbA1c in medically underserved individuals with type 2 diabetes. J Behav Med. (2009) 32:106–17. doi: 10.1007/s10865-008-9188-x 19089606

[25] Shao S Guan J Shen Y. Effects of empowerment theory-based health education combined with gratitude intervention on gratitude level and blood pressure control in elderly hypertensive patients. Psychol Mon. (2024) 19:153–5. doi: 10.19738/j.cnki.psy.2024.14.045

[26] Ding W Zhang Y. Impact of comprehensive health education on blood glucose control and self-efficacy in type 2 diabetes patients. Contemp Med. (2015) 21:3–5.

[27] Zong M Xin X Ni X Jiang Y Li X. Chain mediation effect of self-efficacy and treatment adherence between eHealth literacy and blood pressure control in hypertensive patients. Mil Nurs. (2022) 39:45–48+64.

[28] Venkatesh V Morris MG Davis GB Davis FD. User acceptance of information technology: toward a unified view. MIS Q. (2003) 27:425–78. doi: 10.2307/30036540

[29] Salkovskis PM Rimes KA Warwick HM Clark DM. The health anxiety inventory: development and validation of scales for the measurement of health anxiety and hypochondriasis. Psychol Med. (2002) 32:843–53. doi: 10.1017/S0033291702005822, 12171378

[30] Zhang Y Liu R Li G Mao S Yuan Y. The reliability and validity of a Chinese-version short health anxiety inventory: An investigation of university students. Neuropsychiatr Dis Treat. (2015) 11:1739–47. doi: 10.2147/NDT.S83501, 26213472 PMC4509540

[31] Wallston KA Wallston BS DeVellis R. Development of the multidimensional health locus of control (MHLC) scales. Health Educ Monogr. (1978) 6:160–70. doi: 10.1177/109019817800600107, 689890

[32] Luo L Xie H. Review of self-efficacy assessment scales for geriatric patients with urinary incontinence. Int Urol Nephrol. (2023) 55:2133–8. doi: 10.1007/s11255-023-03661-7, 37330933

[33] Baysan C Yavaş PS. Global research on cyberchondria: scientometric and visual analysis from 2003 to 2022. Stress Health. (2025) 41:e3524. doi: 10.1002/smi.3524, 39698943

[34] Erik H Daniel B Erik A. Development and psychometric properties of the health anxiety behavior inventory (HABI). Behav Cogn Psychother. (2024) 52:616–33. doi: 10.1017/S1352465824000377 39633509

[35] Ali SS Hendawi EN El-Ashry AM Mohammed MS. The relationship between cyberchondria and health literacy among first-year nursing students: the mediating effect of health anxiety. BMC Nurs. (2024) 23:776. doi: 10.1186/s12912-024-02396-9 39434055 PMC11494779

[36] Wang L Zhu R Hou W Zhang C Guo X Wang F. Review of mechanisms underlying health anxiety and health information-seeking behavior. J Med Inform. (2024) 45:16–21.

[37] Feng C Yi M Mo F. Research on interaction mechanisms of multiple subjects in online health communities based on exponential random graph models. Library Inform Serv. (2024) 68:88–101. doi: 10.13266/j.issn.0252-3116.2024.07.009

[38] Fang J Yang Y Wang F. Factors influencing information-sharing willingness of hypertensive patients in health information serendipity contexts. Inform Document Serv. (2024) 45:76–86.

[39] Li Y Wang Y Shi H. Effects of metacognition of health anxiety and family care on fear of disease progression in young and middle-aged cervical cancer patients. J Henan Univ. (2024) 43:225–9. doi: 10.15991/j.cnki.41-1361/r.2024.03.014

[40] Mateusz K Mariusz D. Cyberchondria severity and utilization of health services in polish society: a cross-sectional study. BMC Public Health. (2024) 24:902. doi: 10.1186/s12889-024-18399-9, 38539164 PMC10967182

[41] Du X Lai L Shi C Guo Z Han J Zhang T. Mobile internet-based interpretation bias modification for health anxiety: a randomized controlled trial. Acta Psychol Sin. (2024) 56:1351–71. doi: 10.3724/SP.J.1041.2024.01351

[42] Xie Y. Ethical issues and regulation of health communication on social media. Ethics Res. (2024) 4:132–40. doi: 10.15995/j.cnki.llxyj.2024.04.016

[43] Wu Y. The multiple mediating effects of flow experience between physical exercise and geriatric depression. Chin J Gerontol. (2024) 44:3051–4.

[44] Miller JM Cenzer I Covinsky EK Finlayson E Raue PJ Tang VL. Associations of resilience, perceived control of health, and depression with geriatric outcomes after surgery. Ann Surg. (2024) 10.1097/SLA.0000000000006448 PMC11762354 39056184

[45] Yao L Ren W. Influencing factors, neural basis, and clinical significance of sense of control formation (review). Chin J Health Psychol. (2023) 31:1141–6. doi: 10.13342/j.cnki.cjhp.2023.08.005

[46] Lu L Ji Y. The effect of autonomous choice on subjective well-being: the mediating role of sense of control. Psychol Monthly. (2023) 18:13–6. doi: 10.19738/j.cnki.psy.2023.12.004

[47] Dai W Jiao Q. Silver guardians: research on factors influencing health information adoption among older adults. Sci Commun. (2024) 16:133–9. doi: 10.16607/j.cnki.1674-6708.2024.18.035

[48] Gu C Chen S. Anxious but unable to escape: research on health information cocoons from the perspective of cyberchondria. Mod Inf. (2023) 43:51–63.

[49] Chen X Jiang L Wang H Yang J. How medical staff alleviates job burnout through sports involvement: the mediating roles of health anxiety and self-efficacy. Int J Environ Res Public Health. (2022) 19:11181. doi: 10.3390/ijerph191811181 36141472 PMC9517603

[50] Jin S Li H Shen W Dai W. Research on influencing factors of users' health information-seeking behavior: based on the triadic reciprocal determinism model of social cognitive theory. Inf Sci. (2020) 38:53–61+75. doi: 10.13833/j.issn.1007-7634.2020.06.008

[51] Feng C Meng L Luo G Ji H. Research progress on online health information acquisition behavior among elderly diabetic patients. China Med Pharm. (2024) 14:40–3. doi: 10.20116/j.issn2095-0616.2024.21.10

[52] Zhao W Meng K Ma J Ni Z. Research on factors influencing online health information-seeking behavior based on DEMATEL-ISM: a meta-analysis of empirical studies. J Inf Resour Manage. (2023) 13:53–66+80. doi: 10.13365/j.jirm.2023.02.053

[53] Wang C Lu Z. Analysis of factors influencing rural college students' health information proxy-seeking intention. Inf Res. (2023) 4:24–9.

[54] Stone JK Shafer LA Graff LA Witges K Sexton K Lix LM . The association of efficacy, optimism, uncertainty and health anxiety in inflammatory bowel disease activity over time. J Can Assoc Gastroenterol. (2021) 4:183–4. doi: 10.1093/jcag/gwab002.170

[55] Marr J Wilcox S. Self-efficacy and social support mediate the relationship between internal health locus of control and health behaviors in college students. Am J Health Educ. (2015) 46:122–31. doi: 10.1080/19325037.2015.1023477

[56] Guo Y Zhang Y Tan W. Analysis of the impact of wearable devices on adolescents' exercise habits. Sports Goods Technol. (2024) 3:163–5.

[57] Wang R Zhou C Wu Y Sun M Yang L Ye X . Patient empowerment and self-management behaviour of chronic disease patients: A moderated mediation model of self-efficacy and health locus of control. J Adv Nurs. (2022) 78:1055–65. doi: 10.1111/jan.15077, 34643959

[58] Lilla N Kent N Schulz PJ. Is patient empowerment the key to promote adherence? A systematic review of the relationship between self-efficacy, health locus of control and medication adherence. PLoS One. (2017) 12:e0186458. doi: 10.1371/journal.pone.0186458 29040335 PMC5645121

[59] Yang S. Hard to love: current status and problems of sports participation among adolescents—based on a survey in 10 provinces (cities). China Youth Study. (2020) 7:5–13+61. doi: 10.19633/j.cnki.11-2579/d.2020.0095

[60] Daisy H Oliver M. Perception of COVID-19 threat, low self-efficacy, and external locus of control lead to psychological distress during the COVID-19 pandemic. Psychol Health Med. (2022) 28:1–8. doi: 10.1080/13548506.2022.2124290 36111351

[61] Li S Tang J. Correlation between self-efficacy and perceived health control in elderly hypertensive patients. J Nurs Manag. (2013) 13:13–4.

[62] Capulli E Druda Y Palmese F Butt AH Domenicali M Macchiarelli AG . Ethical and legal implications of health monitoring wearable devices: a scoping review. Soc Sci Med. (2025) 370:117685. doi: 10.1016/j.socscimed.2025.117685, 40010231

[63] Sylvie R Dionne CE Arie N. Self-efficacy and health locus of control: relationship to occupational disability among workers with back pain. J Occup Rehabil. (2011) 21:421–30. doi: 10.1007/s10926-011-9285-5, 21279425

[64] Zhang T Shen Y. "Partner" or "competitor"? The ambivalent role of wearable devices in sports. TV Radio J. (2021) 6:48–52. doi: 10.13994/j.cnki.stj.2021.06.012

[65] Shang M Li H Wan Z Shen R. Model construction and empirical research on overseas users' continuous use intention of smart health wearable devices: a case study of Xiaomi mi band users in South Korea. Math Pract Theory. (2019) 49:9–19.

[66] Chen J Wang T Feng Z Wang H. Research on elderly users' intentions to accept wearable devices based on the improved UTAUT model. Front Public Health. (2023) 11:1035398. doi: 10.3389/fpubh.2023.1035398 PMC9868808 36699866

[67] Pan CC Santis DKK Muellmann S Hoffmann S Spallek J Barnils NP . Sociodemographics and digital health literacy in using wearables for health promotion and disease prevention: cross-sectional nationwide survey in Germany. J Prev Dent. (2024). doi: 10.1007/s10935-024-00821-Y PMC12206174 39692799

[68] Lupton D. The diverse domains of quantified selves: self-tracking modes and dataveillance. Econ Soc. (2016) 45:101–22. doi: 10.1080/03085147.2016.1143726

[69] Stiglbauer B Weber S Batinic B. Does your health really benefit from using a self-tracking device? Evidence from a longitudinal randomized control trial. Comput Human Behav. (2019) 94:131–9. doi: 10.1016/j.chb.2019.01.018

[70] Huckvale K Prieto JT Tilney M Benghozi PJ Car J. Unaddressed privacy risks in accredited health and wellness apps: a cross-sectional systematic assessment. BMC Med. (2015) 13:214. doi: 10.1186/s12916-015-0444-y, 26404673 PMC4582624

[71] Obermeyer Z Powers B Vogeli C Mullainathan S. Dissecting racial bias in an algorithm used to manage the health of populations. Science. (2019) 366:447–53. doi: 10.1126/science.aax2342, 31649194

